# Visceral adiposity index and risks of cardiovascular events and mortality in prevalent hemodialysis patients

**DOI:** 10.1186/s12933-014-0136-5

**Published:** 2014-10-04

**Authors:** Hung-Yuan Chen, Yen-Ling Chiu, Yi-Fang Chuang, Shih-Ping Hsu, Mei-Fen Pai, Ju-Yeh Yang, Yu-Sen Peng

**Affiliations:** Department of Internal Medicine, Far Eastern Memorial Hospital, Division of Nephrology, #21 Nan-Ya South Rd, Section 2, Banciao District New Taipei City, Taiwan; Division of Nephrology, Department of Internal Medicine, National Taiwan University Hospital and National Taiwan University College of Medicine, Taipei, Taiwan; Center for Geriatrics and Gerontology, Taipei Veterans General Hospital, Taipei, Taiwan

**Keywords:** Visceral adiposity index, Cardiovascular complication, Abdominal obesity, Survival

## Abstract

**Background:**

The visceral adiposity index (VAI) is a newly-derived measure of visceral adiposity with well-validated predictive power for cardiovascular (CV) outcomes in the general population. However, this predictability has not been investigated in hemodialysis patients, and whether VAI is superior to waist circumference (WC) and waist-to-height ratio (WHtR) in predicting CV outcomes and survival in hemodialysis patients remains unknown.

**Methods:**

We performed a prospective study including 464 prevalent hemodialysis patients. The composite outcome was the occurrence of death and CV events during follow-up. Using multivariate Cox regression analysis, VAI, WC and WHtR were tested for the predictive power of outcomes. To evaluate the predictive performance of the VAI, WC and WHtR, time-dependent receiver operating characteristic curve (ROC) analysis was performed.

**Results:**

VAI, WC and WHtR positively correlated with each other. Patients with a higher VAI (tertile 3 vs. tertile 1, adjusted hazard ratio (HR), 1.65; 95% confidence interval (CI), 1.12-2.42; tertile 2 vs. tertile 1, adjusted HR, 1.52; 95% CI, 1.1-2.18) had more composite outcomes. VAI had a similar predictive power of all-cause mortality to WC and WHtR, but superior predictive power of composite and CV outcomes to WC when analyzed by a stepwise forward likelihood ratio test. In time-dependent ROC analysis, VAI, WC and WHtR showed similar predictive performance for outcomes.

**Conclusion:**

VAI is an optimal method to measure visceral adiposity to assess long-term CV outcomes and all-cause mortality in prevalent hemodialysis patients. VAI may provide a superior predictive power of CV outcomes to WC and WHtR.

**Trial registration:**

ClinicalTrials.gov NCT01457625

## Introduction

Visceral adiposity is associated with abnormal lipid metabolism, pro-inflammatory activity and insulin resistance in both the general population [1] and dialysis patients [2,3]. Increased visceral adiposity can lead to incident diabetes mellitus (DM) and atherosclerosis in the general population [1,4], and protein-energy wasting in dialysis patients [3,5]. It can also lead to cardiovascular (CV) events and mortality [3,4,6,7]. The linkage between dysfunctional visceral adiposity and CV disease has been proven; however, the best way to measure visceral adiposity in chronic kidney disease (CKD) and dialysis patients remains inconclusive [8]. Waist circumference (WC) [9-13] and waist-to-height ratio (WHtR) [12-14] are commonly used in dialysis patients to assess visceral fat, and the predictability of mortality in hemodialysis (HD) patients further strengthens the role of WC [10]. Nevertheless, the predictive power of WC on all-cause mortality and cardiac death in dialysis patients depends on the body mass index (BMI) [10], and it has recently been reported to be affected by the interaction between WC and triglycerides (TG) and WC and adipokines [15,16]. Machine-based measures of fat mass such as computed tomography [17] and dual energy X-ray absorptiometry [18] are precise and reliable, however, they also are extremely costly and complicated to perform in CKD and dialysis patients.

Recently, a newly-derived anthropometric measure of obesity, the visceral adiposity index (VAI),a sex-specific index based on WC, BMI, TG, and high density lipoprotein cholesterol (HDL-C), has been shown to be an indicator of visceral adipose functionality [19]. It has also been strongly associated with cardiometabolic risks and the prediction of CV outcomes, including coronary artery disease and cerebrovascular disease in the general population [19-21]. However, whether VAI provides superior predictive power for CV outcomes and survival to WC and WHtR remains inconclusive. One landmark study showed that the VAI was better than BMI and WC in the prediction of CV events [19], however, a large-scale population-based study showed that the VAI is less predictive of incident CV disease than other simple anthropometric measures, such as WC or WHtR [22]. In addition, the utility and the significance of the VAI in dialysis patients have yet to be investigated.

The aim of this prospective study was to investigate whether the VAI is a useful tool to assess CV complications and survival, and to test whether the VAI has superior predictive power of outcomes compared to the commonly used anthropometric measures of visceral adiposity in prevalent HD patients.

## Methods and procedures

### Subjects and patients

This is a prospective, observational study performed in two patient cohorts. The first cohort was composed of 370 prevalent patients undergoing maintenance HD [mean age 60 ± 12 years; 162 females; median HD vintage 4.1 years (range: 0.8-19.5 years)]. Information on these patients has been described elsewhere in more detail [23,24]. Among these 370 patients, 347 had complete data on WC, BMI, lipid profiles and high sensitive C-reactive protein (hs-CRP). The second cohort was composed of 216 prevalent HD patients [mean age: 60 ± 12 years; 103 females; median HD vintage 6.1 years (range: 0.6-25.5 years)] [25]. All 216 patients in this cohort had complete data on WC, BMI, lipid profiles and hs-CRP. The exclusion criteria for entry into the current study in both cohorts were: (1) active infection; (2) recent hospitalization within 3 months; (3) psychotic illness or other communication problems; (4) active malignancy; (5) aged less than 20 years; and (6) receiving HD for less than 3 months. There were 99 patients in both the first and second cohort, and therefore 464 patients (mean age:60 ± 12 years; 235 females) who received prevalent HD at the Far Eastern Memorial Hospital, Taiwan, were enrolled from February 2007 (the first cohort) to October 2011 (the 1st participant enrolled in the second cohort) into the analysis. The study design of the first cohort has been reported in the previously published articles [23,24,26,27]. In the second cohort, all subjects gave written informed consent, and the local ethics committees of the involved hospitals approved the study protocol (Far Eastern Memorial Hospital Research Ehics Review Committee, FEMH-IRB-099090-E; chairman Shih-Hong Huang; Oct. 12, 2010; ClinicalTrials.gov; NCT01457625; Oct. 20, 2011). The authors confirm that all ongoing and related trials for these cohorts are registered.

### Measurements of clinical parameters, nutritional and inflammatory status

The demographic data and concurrent medical history of CV disease were recorded. WC was measured at the umbilical level over light clothing, using an un-stretched tape meter, without any pressure to the body surface. BMI was calculated as weight (kg) divided by the square of the height (m^2^). WHtR was calculated as WC (cm) divided by height (cm). Venous blood was sampled in the morning after an overnight fast of more than 8 hours before dialysis.

The nutritional status of the participants was calculated using the geriatric nutritional risk index (GNRI). This index is calculated from serum albumin level and body weight as follows: GNRI = [14.89 × albumin (g/dL)] + [41.7 × body weight/WLo], where WLo is the ideal body weight calculated from the Lorentz equation. The GNRI has been validated in dialysis patients, and a higher GNRI score indicates better nutritional status [28]. We used the immuno-nephelometric method with a Tina-quant CRP (Latex) ultra-sensitive assay (D & P Modular Analyzer, Roche Diagnostics GmbH, Mannheim, Germany) to determine high sensitive C-reactive protein (hs-CRP) levels.

### Visceral Adiposity Index (VAI)

VAI is a sex-specific index based on WC, BMI, TG, and HDL-C, and estimates the visceral adiposity functionality. The VAI was calculated as follows: (TG and HDL-C were in mmol/l and WC in cm) [19].

$$ \mathrm{Male}\ \mathrm{V}\mathrm{A}\mathrm{I} = \left(\mathrm{W}\mathrm{C}/\left[39.68 + \left(1.88*\mathrm{B}\mathrm{M}\mathrm{I}\right)\right]\right)*\left(\mathrm{T}\mathrm{G}/1.03\right)*\left(1.31/\mathrm{H}\mathrm{D}\mathrm{L}-\mathrm{C}\right) $$

$$ \mathrm{Female}\ \mathrm{V}\mathrm{A}\mathrm{I} = \left(\mathrm{W}\mathrm{C}/\left[36.58 + \left(1.89*\mathrm{B}\mathrm{M}\mathrm{I}\right)\right]\right)*\left(\mathrm{T}\mathrm{G}/0.81\right)*\left(1.52/\mathrm{H}\mathrm{D}\mathrm{L}-\mathrm{C}\right) $$

### Outcomes

The outcomes were a composite of all-cause mortality and CV events, considered jointly or separately. The CV events were defined as the new occurrence of CV events including coronary events (non-fatal myocardial infarct, unstable angina and coronary re-vascularization), hospitalized heart failure, incident hospitalized stroke (either ischemic or hemorrhagic stroke), and incident peripheral arterial occlusion disease requiring surgical intervention. The observation period for outcomes was from February 2007 for the patients in the first cohort, from March 2011 for those in the second cohort, and February 2007 for the 99 patients who were recruited in both cohorts. Follow-up was censored on the date of the first CV event, the end of the study (November 1, 2013), the date of death or undergoing renal transplantation, or at the time the patients were transferred to other dialysis facilities and were no longer followed up, whichever came first. Initially, we constructed plots of the VAIs and hazard ratios (HRs) of the outcomes using the Lowess function. The results revealed their non-linear relationship, suggesting the need for stratification of the patients into tertiles according to their VAI scores for outcome analysis. Therefore, we stratified the patients into tertiles according to their VAI score VAI tertile 1 represented the patients who had VAI values within 0.32-1.41, VAI tertile 2 had VAI values 1.42-3.24, and VAI tertile 3 had VAI values 3.25-31.66. The VAI is a gender-specific index, and there has been reported to be a remarkable difference between genders in survival [21,22]. In addition, the VAI, WC and WHtR have been shown to interact with nutritional status in predicting mortality [6,11,12,14,29]. Therefore, we performed pre-specified subgroup analysis (gender and nutritional status) when assessing all-cause mortality.

### Statistical analysis

Continuous data were presented as mean ± SD or median (interquartile range), and categorical data were reported as percentages. Differences in baseline characteristics and biochemical parameters among the patients in tertiles of VAI were compared by ANOVA for continuous variables and the chi-square test for categorical variables. The non-parametric Kruskal-Wallis test was used for non-normally distributed continuous variables.

Outcome analysis was done with using a Cox proportional hazard model, in which the primary predictor variable was either VAI tertile or VAI as continuous variable, and the covariates included age, gender, vintage of HD, presence of DM, hypertension and concurrent CV disease, hemoglobin, calcium phosphate product, hs-CRP, intact parathyroid hormone and nutritional status (GNRI).We also selected WC and WHtR as the primary predictor variables and repeated the outcome analysis. We used the “Enter” method to analyze the hazard ratio of each primary predictor variable in the multivariate Cox regression model, and in order to differentiate the superiority of the predictive power of each primary predictor variable, we also used the “stepwise forward likelihood ratio test” method to analyze the outcomes in the multivariate Cox regression model.

To further evaluate and compare the predictive performance of the VAI, WC and WHtR, we used time-dependent receiver operating characteristic (ROC) curves for censored data, and the area under the ROC curve (AUC) as the criterion. The time-dependent ROC curve estimation was analyzed using open-source statistical software R. All other statistical analyses were performed using SPSS software, version 19.0 (SPSS, Inc., Chicago, IL). A P value of less than 0.05 was considered to be statistically significant.

## Results

### Basic characteristics of all participants and by VAI tertiles

The basic characteristics of all participants are summarized in Table 1. Generally, the patients in VAI tertile 3 had a higher percentage of history of DM, hypertension and concurrent CV disease. In addition, BMI, WC, WHtR, lipid profiles, hs-CRP and GNRI levels were higher in the tertile 3 patients.

**Table 1:** **Baseline characteristics of all patients and patients within VAI tertiles**

	**All patients**	**VAI tertiles**			**P value**
		**Tertile 1 (0.32-1.41)**	**Tertile 2(1.42-3.24)**	**Tertile 3(3.25-31.66)**	
	**N = 464**	**N = 155**	**N = 155**	**N = 154**	
Age (year)	60 ± 12	59 ± 11	57 ± 13	62 ± 11	0.004
Gender (women,%)	51	43	45	64	<0.001
Diabetes mellitus (%)	50	34	47	69	<0.001
Dialysis vintage (years)	3.6 ± 3.7	3.6 ± 3.4	4.0 ± 4.6	3.1 ± 2.8	0.1
History of hypertension (%)	78	70	77	85	0.008
History of previous CVD No. (%)	109 (23)	28 (18)	31 (20)	50 (32)	0.005
Kt/V_urea_	1.6 (1.3, 1.7)	1.5(1.4, 1.7)	1.6 (1.4, 1.7)	1.6 (1.4, 1.6)	0.5
Systolic BP (mmHg)	147 ± 30	139 ± 69	147 ± 50	148 ± 78	0.1
Diastolic BP (mmHg)	85 ± 13	85 ± 26	83 ± 15	86 ± 15	0.1
Body height (cm)	160 ± 8	160 ± 8	160 ± 9	159 ± 9	0.1
Body weight (Kg)	58 ± 12	54 ± 10	59 ± 12	62 ± 13	<0.001
BMI (Kg.m^−2^)	22.7 ± 3.7	21 ± 2.9	22.7 ± 3.3	24.3 ± 4.0	<0.001
Waist circumference (cm)	84.9 ± 11.3	79.3 ± 8.8	85.7 ± 10.5	89.7 ± 11.9	<0.001
VAI	2.17 (1.17,4.12)	0.9(0.69,1.17)	2.19(1.76, 2.71)	5.12(4.12, 7.11)	<0.001
WHtR	0.53 ± 0.07	0.50 ± 0.05	0.53 ± 0.06	0.57 ± 0.07	<0.01
Laboratory data					
Hemoglobin (g/dL)	11.0 ± 1.6	11.1 ± 1.5	11.1 ± 1.6	10.8 ± 1.6	0.1
K (mmol/L)	4.8 ± 0.8	4.9 ± 0.8	4.8 ± 0.8	4.7 ± 0.8	0.1
Ca (mg/dL); corrected	9.2 (8.8, 9.5)	9.0 (8.7, 9.4)	9.2 (8.8, 9.5)	9.3 (8.9, 9.8)	<0.001
P (mg/dL)	5.1 (4.3, 6.2)	5.0 (4.5, 6.2)	5.4 (4.2, 6.4)	5.0 (4.1, 6.0)	0.2
CaxP	47 (38, 56)	45 (39, 56)	49 (39, 59)	47 (38, 56)	0.4
T-CHO (mg/dL)	171 (145,200)	157 (137,192)	161(139, 192)	186(160,211)	<0.001
TG (mg/dL)	135 (92,212)	78 (62,98)	135(116,168)	256(193,321)	<0.001
LDL-C (mg/dL)	89 (67, 115)	81 (66, 108)	89 (68, 116)	97 (68, 118)	0.08
HDL-C (mg/dL)	46 (36, 57)	58 (49, 74)	45(37, 53)	36 (29, 42)	<0.001
iPTH (pg/mL)	234 (118, 438)	257 (131, 513)	238 (131, 423)	198(91, 412)	0.08
hs-CRP (mg/L)	3.4 (1.2, 8.2)	2.0 (0.8, 5.4)	4.1 (1.2, 10.1)	4.6 (2.2, 10.1)	<0.001
Albumin (g/L)	4.1 ± 0.4	4.1 ± 0.4	4.1 ± 0.4	4.0 ± 0.4	0.06
GNRI	103.3 ± 9.7	100.9 ± 8.5	103.6 ± 8.8	105.6 ± 11.0	<0.001
Medications (%)					
ESA	94	93	93	95	0.1
Statins	23	24	22	20	0.1
Anti-hypertensive agents	53	51	51	55	0.2

*Abbreviations*: *VAI* visceral adiposity index, *CVD* cardiovascular disease, *BP* blood pressure, *BMI* body mass index, *WHtR* waist-to-height ratio, *K* potassium, *Ca* calcium, *P* phosphorus, *CaxP* calcium phosphate product, *T-CHO* total cholesterol, *TG* triglyceride, *LDL-C* low density lipoprotein cholesterol, *HDL-C* high density lipoprotein cholesterol, *iPTH* intact parathyroid hormone, *hs-CRP* high-sensitive C-reactive protein, *GNRI* geriatric nutritional risk index, *ESA* erythropoiesis stimulating agents, Statins, HMG-CoA reductase inhibitors.

Note: Conversion factors for units: hemoglobin in g/dL to g/L, ×10; serum calcium in mg/dL to mmol/L, ×0.2495; serum phosphate in mg/dL to mmol/L, ×0.3229; serum T-CHO in mg/dL to mmol/L,×0.02586;serum LDL-C in mg/dL to mmol/L, ×0.02586; serum TG in mg/dL to mmol/L, ×0.01129; serum albumin in g/dL to g/L, ×10. No conversion necessary for serum iPTH in pg/mL and ng/L; serum potassium in mEq/L and mmol/L.

### Composite outcome, all-cause mortality and cardiovascular events

During the follow-up period (median 4.2 years, range 0.3-6.8 years), 219 patients reached the composite outcome; 120 patients died and 162 experienced CV events. In the 162 patients with CV events, 15 had intracranial hemorrhage, 48 had ischemic stroke, 70 had coronary artery disease (either non-fatal acute myocardial infarct or coronary re-vascularization), 20 had peripheral arterial occlusion disease, and 9 were hospitalized for de-compensated heart failure.

In the unadjusted Cox regression model, the patients in VAI tertile 2 (HR, 1.6; 1.13-2.26) and VAI tertile 3 (HR, 2.12; 1.51-2.98) had more composite outcomes. In addition, the patients in VAI tertile 2 (HR, 1.91; 1.26-2.91) and VAI tertile 3 (HR, 2.68; 1.78-4.03) also had more CV outcomes, and the patients in VAI tertile 3 had the worst all-cause mortality (HR, 1.74; 1.11-2.74). Similarly, WC and WHtR also predicted composite and CV outcomes; however, WC and WHtR did not predict all-cause mortality.

In the multivariate adjusted model (Table 2), VAI was a good predictor of composite and CV outcomes. The patients in VAI tertile 3 had 65% and 80% higher risk of having composite and CV outcomes, and the patients in tertile 2 had 52% and 70% higher risk of composite and CV outcomes. However, after adjusting for multiple outcome-related factors, the patients in VAI tertile 3 had a marginally higher risk of mortality (HR, 1.49; 1.0-2.5, P = 0.06). Similarly, a 10-cm larger WC was associated with a 29% and 36% higher risk, and a 0.01 unit increase in WHtR was associated with 5% and 6% higher risk for composite and CV outcomes after multivariate adjustments. However, WC and WHtR did not predict all-cause mortality after adjustments (Table 2).

**Table 2:** **VAI, WC, WHtR and baseline factors associated with outcomes in all participants analyzed by Cox proportional-hazards regression model with multivariate adjustments (with the Enter method)**

**Variables**	**Composite outcome**		**Cardiovascular outcome**		**All-cause mortality**	
	**Adjusted HR (95% CI)** ^**§**^	**P**	**Adjusted HR (95% CI)** ^**§**^	**P**	**Adjusted HR (95% CI)** ^**§**^	**P**
VAI (tertile 2 vs. tertile 1)	1.52 (1.1-2.18)	0.02	1.70 (1.1-2.61)	0.02	1.33(0.81-2.16)	0.3
VAI (tertile 3 vs. tertile 1)	1.65 (1.12-2.42)	0.01	1.80 (1.1-2.8)	0.01	1.49(1.0-2.5)	0.06
VAI (every 1 unit increase)	1.38 (1.08-1.98)	0.002	1.4 (1.11-1.89)	0.01	1.21 (0.99-1.29)	0.4
WC (every 10 cm increase)	1.29 (1.08-1.54)	0.005	1.36 (1.1-1.67)	0.004	1.11(0.88-1.4)	0.4
WHtR (every 0.01 unit increase)	1.05(1.02-1.08)	0.003	1.06(1.02-1.09)	0.003	1.02(0.98-1.06)	0.4
**Demographic data**						
With DM	2.2 (1.63-3.0)	<0.001	2.44 (1.5-3.5)	<0.001	2.05(1.36-3.08)	0.001
With CV disease history	1.48 (1.1-2.0)	0.01	1.83 (1.31-2.57)	<0.001	1.37(0.92-2.06)	0.1
With HTN	1.13 (0.77-1.67)	0.5	1.31 (0.82-2.1)	0.3	1.01(0.5-1.31)	0.4
Age (every 10 years increase)	1.38 (1.24-1.52)	<0.001	1.28 (1.1-1.4)	0.001	1.57(1.37-1.77)	<0.001
HD vintage (every 1 year increase)	1.02 (0.98-1.06)	0.3	1.03 (0.98-1.08)	0.2	1.01(0.95-1.07)	0.8
**Laboratory parameters**						
hs-CRP	1.05 (0.99-1.12)	0.08	1.04 (0.96-1.12)	0.4	1.1(1.02-1.18)	0.01
Hemoglobin	0.9 (0.82-0.99)	0.05	0.93 (0.83-1.05)	0.3	0.92(0.8-1.05)	0.2
GNRI	0.98 (0.96-0.99)	0.002	0.99 (0.97-1.01)	0.4	0.96(0.94-0.98)	<0.001
iPTH	1.0 (0.99-1.01)	0.2	1.0 (0.99-1.02)	0.4	1.0(1.0-1.001)	0.8
CaxP	1.003 (0.99 ~ 1.01)	0.5	1.002(0.99-1.01)	0.7	1.008(0.99-1.07)	0.2

*Abbreviations*: *VAI* visceral adiposity index, *WC* waist circumference, *WHtR* waist-to-height ratio, *HR* hazard ratio, *CI* confidence interval, *P* P value, *DM* diabetic mellitus, *CV* cardiovascular, *HTN* hypertension, *HD* hemodialysis, *hs-CRP* highly-sensitive C-reactive protein, *GNRI* geriatric nutritional risk index, *iPTH* intact parathyroid hormone, *CaxP* calcium phosphate product.

^§^Adjusted for gender, age, HD vintage, presence of DM, HTN and concurrent CV disease, hemoglobin, iPTH, hs-CRP, calcium phosphate product and GNRI levels.

### The predictive performance of the VAI, WC and WHtR on the composite outcomes, cardiovascular events and all-cause mortality

We used the stepwise forward likelihood ratio method in the adjusted multivariate model (Table 3), and found that VAI was a good predictor of composite and CV outcomes. However, WC and WHtR did not predict composite outcome, CV outcome or all-cause mortality, and the VAI and WHtR were also not good predictors of all-cause mortality.

**Table 3:** **VAI, WC and WHtR in all participants analyzed by Cox proportional-hazards regression model with multivariate adjustments (with the stepwise forward likelihood ratio method)**

**Variables**	**Composite outcome**		**Cardiovascular outcome**		**All-cause mortality**	
	**Adjusted HR (95% CI)** ^**§**^	**P**	**Adjusted HR (95% CI)** ^**§**^	**P**	**Adjusted HR (95% CI)** ^**§**^	**P**
VAI (tertile 2 vs. tertile 1)	1.5 (1.07-2.08)	0.04	1.72 (1.13-2.63)	0.01	-	0.8
VAI (tertile 3 vs. tertile 1)	1.62 (1.22-2.32)	0.04	1.69 (1.11-2.6)	0.02	-	0.4
WC (every 10 cm increase)	-	0.4	-	0.3	-	0.1
WHtR (every 0.01 unit increase)	-	0.1	-	0.2	-	0.2
**Taking VAI as a continuous variable**						
VAI (every 1 unit increase)	1.4 (1.23-2.25)	0.01	1.69 (1.3-2.2)	0.01	-	0.4
WC (every 10 cm increase)	-	0.4	-	0.3	-	0.1
WHtR (every 0.01 unit increase)	-	0.1	-	0.2	-	0.2

*Abbreviations*: *VAI* visceral adiposity index, *WC* waist circumference, *WHtR* waist-to-height ratio, *HR* hazard ratio, *CI* confidence interval, *P* P value, *DM* diabetic mellitus, *CV* cardiovascular, *HTN* hypertension, *HD* hemodialysis, *hs-CRP* highly-sensitive C-reactive protein, *GNRI* geriatric nutritional risk index, *iPTH* intact parathyroid hormone, *CaxP* calcium phosphate product.

^§^Adjusted for gender, age, HD vintage, presence of DM, HTN and concurrent CV disease, hemoglobin, iPTH, hs-CRP, calcium phosphate product and GNRI levels.

-: indicated non-selected in the stepwise likelihood ratio model.

### Time-dependent ROC analysis of the VAI, WC and WHtR as predictors of composite outcome, cardiovascular outcome and all-cause mortality

The AUC for the VAI, WC and WHtR versus outcomes are shown in Table 4. VAI, WC and WHtR had similar predictive performance for all aspects of outcome analysis at the fourth, fifth and sixth years of follow-up.

**Table 4:** **AUCs of ROC curves for the prediction of composite outcome, CV outcome and all-cause mortality by VAI, WC and WHtR**

	**VAI**	**WC**	**WHtR**	**P-value (compare WC and VAI)**	**P-value (compare WHtR and VAI)**
Composite outcomes					
T = 4 years	60.85 ± 2.87	59.26 ± 3.09	59.98 ± 3.07	0.63	0.79
T = 5 years	59.67 ± 2.97	60.69 ± 3.09	60.79 ± 3.08	0.76	0.73
T = 6 years	62.82 ± 3.01	61.83 ± 3.17	62.42 ± 3.14	0.77	0.90
CV outcomes					
T = 4 years	63.12 ± 3.06	62.23 ± 3.38	63.26 ± 3.36	0.80	0.97
T = 5 years	61.76 ± 3.16	63.06 ± 3.28	62.97 ± 3.29	0.71	0.73
T = 6 years	65.56 ± 3.15	64.31 ± 3.33	64.65 ± 3.31	0.72	0.79
All-cause mortality					
T = 4 years	57.41 ± 3.51	51.87 ± 3.91	51.44 ± 3.78	0.22	0.15
T = 5 years	57.41 ± 3.51	54.74 ± 3.62	54.91 ± 3.56	0.74	0.76
T = 6 years	57.08 ± 3.28	57.56 ± 3.54	58.16 ± 3.49	0.90	0.76

Note: Values are expressed as AUC ± SE (95% confidence interval).

*Abbreviations*: *AUC* area under curve, *ROC* receiver operating characteristic, *CV* cardiovascular, *VAI* visceral adiposity index, *WC* waist circumference, *WHtR* waist-to-height ratio.

### Pre-specified subgroup analysis of the impacts of the VAI, WC, and WHtR on all-cause mortality

**Figure 1:**
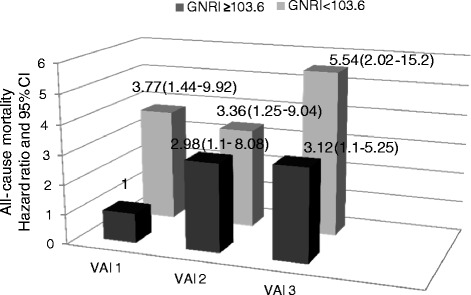
**Relationship between visceral adiposity index (VAI) tertiles and all-cause mortality in hemodialysis patients with different nutritional status.** The VAI predicted all-cause mortality in patients with a better nutritional status (GNRI ≥ 103.6) but not in those with a worse nutritional status (GNRI < 103.6). Analysis were adjusted for gender, age, hemodialysis vintage, presence of diabetes, hypertension and concurrent cardiovascular disease, hemoglobin, intact parathyroid hormone, high sensitive C-reactive protein and calcium phosphate product.

In men, the VAI (VAI 3 versus VAI 1, HR, 2.95; 1.3-6.69 and VAI 2 versus VAI 1, HR, 2.25; 1.15-4.39), WC (HR, 1.51; 1.13-2.03, every 10 cm increase) and WHtR (HR, 1.09; 1.03-1.16, every 0.01 unit increase) all predicted all-cause mortality. However, in women, the VAI, WC and WHtR did not predict all-cause mortality. The VAI predicted all-cause mortality in patients with better nutritional status (GNRI ≥ 103.6) but not in patients with worse nutritional status (GNRI < 103.6) (Figure 1). However, WC did not predict all-cause mortality in either the patients with better (GNRI ≥ 103.6, HR = 1.12; 0.67-2.45, every 10 cm increase) or worse (GNRI < 103.6, HR = 1.03; 0.58-2.55) nutritional status. Similarly, WHtR did not predict all-cause mortality in either patients with better (HR = 1.06; 0.55-1.7, every 0.01 unit increase) or worse (HR = 1.01; 0.38-3.55, every 0.01 unit increase) nutritional status.

## Discussion

The main findings of this study are that in prevalent HD patients, the VAI could predict CV and composite outcomes as well as all-cause mortality in men and in those with a better nutritional status. Moreover, the VAI might have a better predictive performance on composite outcome and CV outcome than WC and WHtR in HD patients.

Abdominal obesity is a crucial factor when predicting CV complications, and is a major component of the metabolic syndrome in both the general population and diabetic patients [7]. In our previous cohort [25] and in another study [10], nearly 40 ~ 50% of dialysis patients had abdominal obesity according to the diagnostic criteria recommended by the US National Cholesterol Education Programme Adult Treatment Panel III (NCEP ATP III) guidelines. Associations between abdominal obesity, inflammation, atherosclerosis and consequently CV mortality and morbidity have also been reported in both dialysis [3,5,10,30] and CKD patients [31,32]. Therefore, it is worthwhile to precisely measure the severity of abdominal obesity in dialysis patients.

In this study, the HD patients with higher abdominal adiposity as measured by the VAI, had worse lipid profiles, higher hs-CRP levels (Table 1) and more importantly, higher CV and composite outcomes. The predictive value of the VAI for CV complications and cardiometabolic risks has been validated in the general population [19,21,22] and in some diseases such as non-alcoholic fatty liver disease [33], acromegaly [34] and sleep apnea syndrome [35].To the best of our knowledge, no investigation has explored the predictive power of the VAI on CV outcomes and survival in dialysis patients, and that this is the first study to clearly show that higher VAI values predicted worse composite outcomes (Table 2).This validation in HD subjects is worth emphasizing because many important CV outcomes and survival predictors in the general population such as BMI and lipid profiles have an inverse predictive power in dialysis subjects. Therefore, our results confirm that the VAI is also a reliable tool to assess abdominal adiposity dysfunction in dialysis patients, and to predict consequent cardiometabolic complications.

WC and WHtR are commonly used as measures of abdominal adiposity in the general population and dialysis subjects because they are easy to calculate, have been validated as the gold standard of visceral adiposity measures, and the correlation with CV outcomes has been established [8,10,11,14]. However, there are drawbacks of WC-based assessments of abdominal adiposity. First, WC represents both visceral adipose tissue and subcutaneous adipose tissue, which correspond to different outcome results [36,37]. Second, WC correlates poorly with changes in visceral adiposity over time in CKD patients [38]. Third, although the predictive power of all-cause mortality and incident CV events in HD patients has been validated, the predictive power only occurs after adjusting for BMI, which implies that an interaction between WC and BMI explains the outcomes [10] The same investigator also identified interactions between WC versus TG levels and WC versus adipokine levels when assessing all-cause and CV mortality [15,16] in HD patients. Therefore, WC may be an imperfect measure of visceral adiposity in dialysis patients, and therefore other tools to measure visceral adiposity should be carefully considered. The VAI is also a WC-based measure, however it is adjusted for BMI and interactions with TG and HDL-C. Therefore, the VAI may be a better tool for measuring visceral adiposity and in assessing its impacts on CV outcomes and survival. In our multivariate Cox regression results, only VAI tertile 3 predicted a marginally higher risk of mortality, whereas WC and WHtR did not (Table 2). Further, when we applied the stepwise forward likelihood ratio method, the VAI had a better predictive performance than WC and WHtR (Table 3). In time-dependent ROC analysis, the VAI had a similar AUC to those of WC and WHtR in the prediction of all outcomes (Table 4). These results further strengthen the hypothesis that the VAI may be a superior predictor, or at least, an equal predictor compared with WC and WHtR when assessing the impact of visceral adiposity dysfunction on CV outcomes and mortality.

Over-nutrition leads to abdominal obesity and consequently incident diabetes and CV events [4]. In addition, nutritional status has been shown to be a determinant of survival in dialysis patients [39]. In our results, the patients with higher VAI values had better nutritional status (Table 1), and therefore we presumed an interaction between the VAI and nutritional status when assessing the predictive power for all-cause mortality. As shown in Figure 1, higher VAI values predicted a higher risk of mortality in the HD patients with better nutritional status (GNRI ≥ 103.6), but not in those with a worse nutritional status. We hypothesize that dialysis patients with a better nutritional status are exempt from the impacts of malnutrition and protein-energy wasting on overall survival, and therefore the impact of visceral adiposity on all-cause mortality becomes pronounced. In contrast, in patients with a relatively poor nutritional status, higher visceral adiposity predicts a higher likelihood of survival, a phenomenon called obese sarcopenia, which has been explored in dialysis patients [5,6]. Hence, the impact of the VAI on all-cause mortality would be less in this circumstance.

The strengths of this study are its prospective nature, optimal observational period, full adjustment for multiple CV and survival factors in outcome analysis, and extended outcome analysis including both CV complications and all-cause mortality. However, there are some limitations to this study. First, we did not perform other machine-based assessments of visceral adiposity such as computed tomography or dual energy X-ray absorptiometry to validate the role of the VAI in measuring visceral adiposity in HD patients. Second, the annual mortality rate in this study was similar to our previously report [26] and to another study from Taiwan [40] in dialysis patients, but lower than reported in Western counties [10]. Furthermore, 34 patients (6.9%) in this study were censored because they were transferred to other dialysis facilities and were no longer followed up, and therefore the annual mortality rate may have been underestimated and the results of the consequent Cox survival analysis may be inaccurate. Third, the present study was based in a single center cohort in Taiwan, and therefore further investigations in multiple centers or in different ethnicities are warranted.

In summary, our results suggest that the VAI positively correlates with WC and WHtR and is an optimal method to measure visceral adiposity to assess long-term CV outcomes and all-cause mortality in prevalent HD patients. In HD patients with a good nutritional status, the VAI has a superior predictive power for all-cause mortality than WC or WHtR.
